# Ranaviruses: Not Just for Frogs

**DOI:** 10.1371/journal.ppat.1003850

**Published:** 2014-01-16

**Authors:** V. Gregory Chinchar, Thomas B. Waltzek

**Affiliations:** 1 Department of Microbiology, University of Mississippi Medical Center, Jackson, Mississippi, United States of America; 2 Department of Infectious Diseases and Pathology, College of Veterinary Medicine, University of Florida, Gainesville, Florida, United States of America; University of Florida, United States of America

## Introduction

Ranaviruses (family *Iridoviridae*) were discovered serendipitously during an attempt to generate frog kidney cell cultures for the propagation of Lucke herpesvirus, an oncogenic herpesvirus [1]. Unexpectedly, some cultures displayed spontaneous cytopathic effect suggestive of a viral infection. Frog virus (FV)-1 and -2 were recovered from normal kidneys, whereas FV3, isolated from a frog with renal carcinoma, became the focus of further study. A number of “intriguing” features, i.e., a highly methylated genome, ability to rapidly turn-off host macromolecular synthesis, a circularly permuted/terminally redundant genome, the use of both host- and virus-encoded RNA polymerases, and absence of polyadenylated mRNA [2], fueled early FV3 study and provided insight into a poorly characterized family of nuclear, cytoplasmic, large DNA-containing viruses. However, because ranaviruses were not pathogenic for humans or commercially important animals, were not, as originally thought, oncogenic, and did not appear to have adverse long-term impacts on wildlife, FV3 research remained a viral backwater. Recent years have seen renewed interest in ranaviruses and other iridoviruses because they have been increasingly linked to die-offs, often marked, among cultured and wild fish, amphibians, and reptiles. Here, we review ranavirus replication and gene function, then focus on their impact on cold-blooded vertebrates.

## 1. Replication and Gene Function

**Figure 1:**
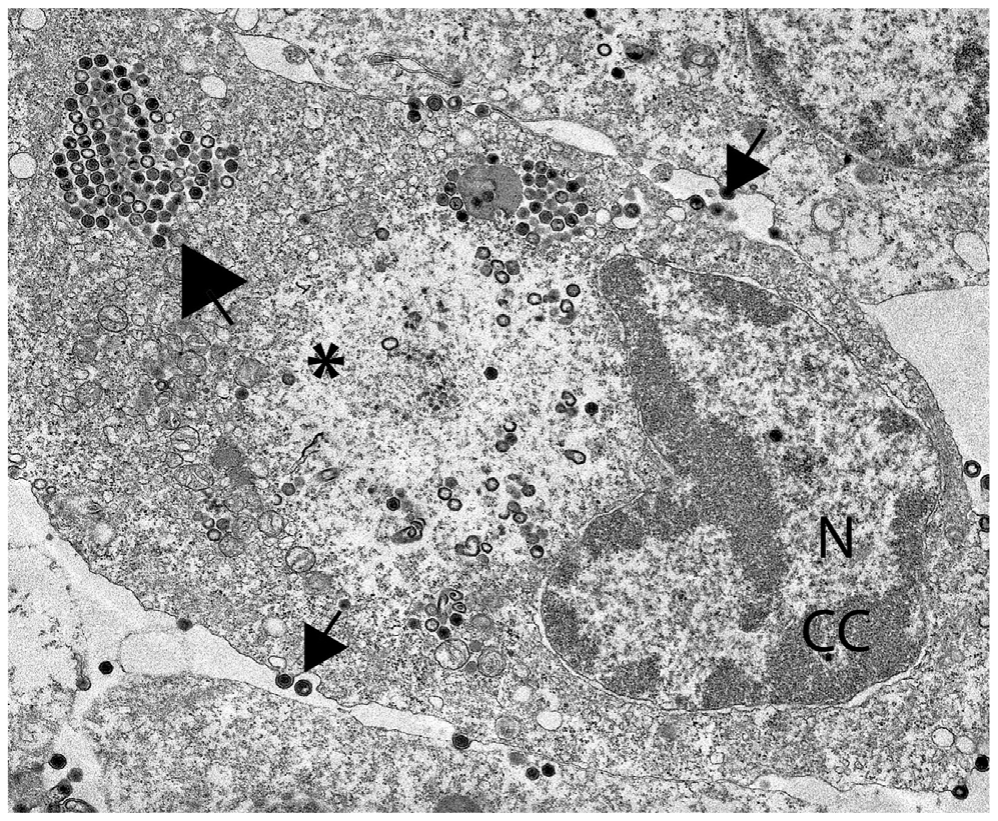
FV3-infected fathead minnow (FHM) cells. FHM cells were infected at the permissive temperature with FV3 temperature sensitive mutant ts12814 at a multiplicity of infection of 5 PFU/cell, and processed for electron microscopy at 24 hr post infection. Small arrows indicate virions budding from the plasma membrane; large arrow indicates a small paracrystalline array. N, nucleus; CC, chromatin condensation indicative of apoptosis; *, viral assembly site.

FV3 is the type species and best characterized member of the genus *Ranavirus*, one of five genera within the family *Iridoviridae* [3]. For both historical and technical reasons, ranavirus replication has been elucidated using primarily FV3 as a model [2], [4]. Replication involves both nuclear and cytoplasmic compartments and utilizes both host and viral enzymes. Early viral gene expression takes place in the nucleus and is catalyzed by host RNA polymerase II. Unit-length viral genomes are synthesized within the nucleus using a virus-encoded DNA polymerase and are subsequently transported to the cytoplasm where they are methylated and linked into large concatamers. Late viral transcription likely occurs in the cytoplasm and is catalyzed by a transcriptase that contains at least two virus-encoded homologs of RNA polymerase II (vPOL II). Characteristic icosahedral virions form in large, morphologically distinct assembly sites and are seen scattered throughout assembly sites, within paracrystalline arrays, or budding from the plasma membrane (Figure 1).

Although the outlines of ranavirus replication are known, the precise roles that specific viral genes play remain to be elucidated. Ranavirus genomes encode 100–140 proteins [2]. The functions of about a third have been inferred by homology or biochemical/genetic study, whereas that of the remaining genes, which match only genes found in other family members, are unknown. Recent studies using recombinant proteins [4], [5], antisense morpholino oligonucleotides (asMO) [6], and knock out [7], [8] mutants have determined the function of several viral proteins. For example, the role of the viral homolog of eukaryotic translational initiation factor eIF-2α (vIF-2α) in maintaining protein synthesis in the face of virus-mediated shut-off was confirmed using recombinant [5] and genetic approaches [7], [8], and the role of vPOL II in late gene transcription was demonstrated following knock down using an asMO [6]. Future studies will ascertain the roles not only of viral replication proteins, but also those that play putative roles in immune evasion, host range, and virulence. Identifying virulence genes is critical because this may provide insight into elements of host immunity critical for survival and, following their knock out, permit development of attenuated viruses as vaccines.

## 2. Ranaviruses Provide Insight into Antiviral Immunity among Ectothermic Vertebrates

Experimental infection of *Xenopus laevis* by FV3 offers an excellent opportunity to elucidate the role of the amphibian immune system in disease protection and to understand the evolutionary origins of vertebrate immunity. This model couples FV3, the best-characterized ranavirus, with *Xenopus laevis*, an amphibian with the most fully characterized immune system [9]. Using it, Robert and colleagues confirmed that adult frogs, although susceptible to FV3, confine infection to the kidney and rapidly resolve it using both antiviral antibodies and cytotoxic T cells [10]–[12]. In contrast, tadpoles and immunocompromised adults develop widespread systemic infections that often result in death. FV3 also infects macrophages, which may suppress immunity, and results in a low percentage of macrophages that appear to become persistently infected [13]. Furthermore, microarray analysis of FV3-infected fathead minnow cells indicated that viral infection triggered the induction of multiple immune-related genes that, similar to their mammalian counterparts, likely play critical roles in antiviral immunity (Chinchar, Cheng, Garcia-Reyero, unpublished). Lastly, studies of putative virus-encoded immune evasion proteins may provide insight into elements of host immunity critical for survival.

## 3. Ranaviruses: Promiscuous Pathogens of Cold-Blooded Vertebrates

In contrast to lymphocystiviruses and megalocytiviruses, two genera within the family *Iridoviridae* that infect only bony fish, ranaviruses target three taxa of ectothermic vertebrates: amphibians, reptiles, and bony fish. Although ranaviruses such as *Santee-Cooper Ranavirus* and *Epizootic Haematopoietic Necrosis Virus* (EHNV) target animals of a single class, e.g., fish, FV3 and FV3-like viruses infect and cause disease in frogs, turtles, and fish [2]. Supporting this view, the genome of softshell turtle iridovirus was shown to be nearly identical to FV3, including a unique truncation within the *vIF-2α* gene [14]. This ability to infect animals from diverse taxonomic classes, while not unique to ranaviruses, may play a role in the persistence of ranaviruses in the environment and the spread of ranaviruses to other species and geographic regions.

## 4. Ranavirus Infections Are Common among Wild Amphibians and Reptiles

Appreciation of ranaviruses as contributors to morbidity and mortality among ectotherms has markedly increased. Early disease reports were sporadic, but hinted at the potential impact such infections had on wildlife, especially endangered or geographically constrained populations. As diagnostic tools improved, die-offs that had been attributed to nonpathogenic causes, “other” infectious agents, or listed as “unknown” were increasingly shown to involve ranaviruses. Thus infection of box turtles in the Eastern United States [15], tiger salamanders in western North America [16], wild and captive boreal toads (Chinchar, Waltzek, and Pessier, unpublished), and common frogs in the United Kingdom [17] were all due to ranavirus infection. Indicative of their impact, the majority of recent amphibian mortality events have been attributed to ranaviruses [18], [19]. Ranavirus prevalence varies with the specific host species, location, and season and ranges from 0% to over 80%. However, although prevalence may be high in a given population, the specific host, viral, and environmental factors that turn subclinical infections into life-threatening ones are poorly understood. In addition, the earlier view that ranaviruses were geographically clustered has been shattered by studies showing FV3 and FV3-like viruses in North and South America as well as Europe and Asia. It is thought that the worldwide movement of animals coupled with the broad host range of these viruses may be responsible for their geographic dispersion.

## 5. Ranaviruses: Emerging Threats to Global Aquaculture

EHNV, the first ranavirus shown to induce lethal systemic disease in fish, has negatively impacted rainbow trout farms in southeastern Australia since 1986 [20]. Similarly, *European Catfish Virus* has resulted in periodic high mortality epizootics among cultured European catfish including sheatfish, brown bullheads, and black bullheads. Likewise grouper mariculture in Asia has experienced significant economic losses as a result of infections with Singapore Grouper Iridovirus and Grouper Iridovirus, and epizootics of FV3-like viruses have been reported among cultured sleepy gobies in Thailand [21] and softshell turtles in China [14]. Epizootics attributed to FV3-like viruses within ranaculture facilities rearing a variety of frogs and anurans in North America, South America, Europe, and Asia further illustrate the global threat of ranaviruses [22]. Ranaviruses have impeded efforts to restore stocks of critically endangered wildlife including pallid sturgeon within the Missouri River basin of the United States and the Chinese giant salamander distributed across central and southern China. High mortality epizootics were reported among young-of-the-year pallid sturgeon in 2001, 2009, and 2013 at the Blind Pony Hatchery in Sweet Springs, Missouri (Waltzek, unpublished). Likewise, high mortality epizootics have been reported among Chinese giant salamanders of all age classes on multiple farms in 2010 and 2011 [23]. In both cases, FV3-like ranaviruses were isolated from moribund sturgeon and salamanders and induced lethal disease within their respective hosts. The apparent increase in ranavirus epizootics among aquatic animals reared for food, or as part of restoration programs, underscores the need for improved biosecurity practices and a better understanding of the pathogen-host-environment imbalance often created under artificial culture conditions.

The promiscuous nature of ranaviruses coupled with the global trade of live animals indicates the importance of updating regulatory policies aimed at mitigating the impact of these emerging pathogens on aquaculture and imperiled wildlife. Although the World Organization for Animal Health currently requires reporting ranavirus-associated amphibian epizootics and EHNV outbreaks in fish, FV3-like viruses detected in wild fish or turtles are not reportable despite the presence of genetically identical ranaviruses in epizootics involving sympatric ectothermic vertebrates [2]. Continued monitoring will provide a more complete view of the prevalence and impact of ranavirus infections on wildlife, and molecular studies will identify genes critical for viral replication and immune evasion. Together they may allow us to better protect both wild and cultured ectothermic vertebrates.
